# Role of DNA methylation in the dietary restriction mediated cellular memory

**DOI:** 10.1007/s11357-017-9976-8

**Published:** 2017-05-05

**Authors:** Archana Unnikrishnan, Jordan Jackson, Stephanie A. Matyi, Niran Hadad, Benjamin Wronowski, Constantin Georgescu, Karla P. Garrett, Jonathan D. Wren, Willard M. Freeman, Arlan Richardson

**Affiliations:** 1 Department of Geriatric Medicine, University of Oklahoma Health Sciences Center, Oklahoma City, OK USA; 2 Reynolds Oklahoma Center on Aging, University of Oklahoma Health Sciences Center, Oklahoma City, OK USA; 3 Department of Physiology, University of Oklahoma Health Sciences Center, Oklahoma City, OK USA; 4 Department of Biochemistry and Molecular Biology, University of Oklahoma Health Sciences Center, Oklahoma City, OK USA; 5 Arthritis and Clinical Immunology Program, Oklahoma Medical Research Foundation, Oklahoma City, OK USA; 6 Oklahoma City VA Medical Center, Oklahoma City, OK USA

**Keywords:** Dietary restriction, Gene expression, DNA methylation, Transcriptome, Epigenetics, Stem cells

## Abstract

An important facet of dietary restriction (DR) that has been largely overlooked is that DR can have early effects that create a cellular memory, which persists even when DR is discontinued. The goal of this study was to determine if DNA methylation played a role in the cellular memory of DR by examining the effect of short-term DR on gene expression and DNA methylation and determining if the changes in expression and DNA methylation persist when DR is discontinued and mice returned to ad libitum (AL) feeding. We show that DR can induce substantial changes in gene expression within 1 month of its implementation in various tissues, and more interestingly, ~19–50% of these changes in gene expression persist across the tissues even when DR is discontinued. We then determined whether DR induced changes in DNA methylation in the promoter of three candidate genes identified from our gene expression analysis (*Pomc*, *Hsph1*, and *Nts1*) that correlated with the changes in the expression of these genes. Decreased methylation at three specific CG sites in the promoter of the *Nts1* gene encompassing the distal consensus AP-1 site was correlated with increased Nts1 expression. Both the promoter hypomethylation and increased *Nts1* expression persisted even after DR was discontinued and mice fed AL, supporting our hypothesis that DNA methylation could play a role in the memory effect of DR. The changes in DNA methylation in the *Nts1* gene are likely to occur in intestinal stem cells and could play a role in preserving the intestinal stem cell pool in DR mice.

## Introduction

DNA methylation is a molecular mechanism that has evolved to provide cellular memory to cells/organisms. Cytosines, especially those contained in CG dinucleotide motifs, are targeted for methylation (5mC) by DNA methyltransferases. Functionally, 5mC can indirectly regulate chromatin status via cross talk with histone modifications (Hashimshony et al. 2003; Eden et al. 1998) and directly affect the ability of transcription factors and other DNA-binding proteins to access DNA. McGhee and Ginder (1979) were the first to suggest that DNA methylation controlled gene expression, and Jones and Taylor (1980) showed that 5-azacytidine (an inhibitor of DNA methyltransferases) prevented the differentiation of cells by altering the expression of the genes involved in differentiation. In the context of gene promoters, hypomethylated CGs are generally associated with active, constitutively expressed genes, while hypermethylated CGs are generally associated with lowly expressed/silenced genes (Bird 2002). 5mC can be both a persistent molecular signal, which lasts the lifetime of a cell, and be passed on to daughter cells (Holliday 2006). Taken together, it is now accepted that DNA methylation can regulate the expression of specific genes and thereby potentially affect the physiological function of a cell/tissue over the lifetime of the organism.

The potential role of DNA methylation in aging was suggested in the early 1980s by Wilson and Jones (1983) when they reported a decrease in 5mC content of DNA in late passage fibroblasts. It was argued that genome-wide hypomethylation would lead to random loss of silencing resulting in the inappropriate expression of genes in tissues/cells as an organism aged (Pogribny and Vanyushin 2010). However, it is now obvious that the location of specific 5mC residues in the genome is critical to their impact on gene expression. Only recently has the technology been available to interrogate the whole genome with respect to changes in DNA methylation at specific sites. Furthermore, DNA methylation at 353 CG sites in the human genome has been shown to be a quantitative biomarker of chronological aging (Hannum et al. 2013; Horvath 2013; Weidner et al. 2014). Although these data tell us little about how the changes in DNA methylation impact aging at the molecular level, they provide strong evidence of a link between DNA methylation and aging. Furthermore, studies show that epigenetic dysregulation, specifically DNA methylation, in tissues such as liver and adipose tissue in response to age or inutero malnutrition can potentially lead to physiological and transcriptional changes normally associated with aging (Thompson et al. 2010; Heo et al. 2016).

Over the past 50 years, DR has been shown to extend the lifespan of a wide variety of organisms, ranging from invertebrates to mammals, such as rodents (Weindruch et al. 1986), dogs (Kealy et al. 2002), and nonhuman primates (Colman et al. 2014). DR has also been shown to delay the onset of most age-related diseases and improve most physiological processes that decline with age. An important aspect of DR that has largely been overlooked is that DR can have early effects that create a cellular memory which persists throughout life even when DR is discontinued. Yu et al. (1985) showed that 4.5 months of DR (from 6 weeks to 6 months of age) significantly increased the median (13%) and maximum (10%) lifespan of rats. More recently, Miller’s group studied the effect of restricting food consumption preweaning by increasing litter size 50%, from 8 to 12 mice per litter (Sun et al. 2009). The mice were fed ad libitum (AL) the rest of their life after weaning. Mice raised 12 pups per litter lived 18% longer than the mice raised eight pups per litter. DR has also been shown to alter physiological processes that persist even when DR is discontinued, e.g., insulin sensitivity, which is a hallmark feature of DR. Mice fed DR retained a metabolic memory in terms of improved glucose tolerance after DR was discontinued (Cameron et al. 2012; Selman and Hempenstall 2012).

Because DR has early effects that can lead to increased longevity even when DR is discontinued, we hypothesize that epigenetic changes induced within months after the initiation of DR alter the transcriptome in such a way that physiological functions are modified over the lifespan of an organism, resulting in an increase in lifespan. To test this hypothesis, we first determined whether short-term DR altered gene expression and if these changes persisted when DR was discontinued. Secondly, we used the novel bisulfite amplicon sequencing (BSAS) technology (Masser et al. 2013) to measure DNA methylation in specific CG sites in the promoters of several genes. Our data show that short-term DR alters the transcripts of a large number of genes in various tissues, and ~19–50% of these changes persist in total across the tissues when DR is discontinued. Furthermore, we show that short-term DR significantly alters DNA methylation in the promoter of a gene, *Nts1*, that is correlated with the changes in the expression of the gene induced by DR.

## Methods

### Animals and feeding regiment

Male C57BL/6 mice were purchased from the Jackson laboratory (Bar Harbor, ME) and housed in the animal facility at the University of Oklahoma Health Sciences Center and maintained under SPF conditions in a HEPA barrier environment. The animals were fed irradiated NIH-31 mouse/rat diet from Teklad (Envigo, Madison, WI) until 4 months of age. In the first group of mice, the mice were separated into two dietary regimens at 4 months of age: ad libitum (AL, *n* = 5) and DR (*n* = 10). The DR group was fed 60% of the food consumed by the AL animals for 1 month. After a month of DR, five animals from the DR group were fed AL for 2 months (DR-AL). Mice were then sacrificed and tissues harvested (epididymal white adipose tissue, liver, colon (intestinal mucosa), hypothalamus, and hippocampus), snap frozen in liquid nitrogen, and stored at −80 °C until used. These tissues were used for RNA isolation and RNA-Seq analysis.

In the second set of mice, gene expression and DNA methylation analyses were measured in three groups of mice: 4-month-old mice fed AL (*n* = 5), mice fed DR (60% AL, *n* = 10) for 4 months starting at 4 months of age, and five animals from the DR group that were switched to AL feeding and continued for 5 months (DR-AL). Mice were then sacrificed and tissues harvested, snap frozen in liquid nitrogen, and stored at −80 °C until used. These tissues were used for RNA/DNA co-isolation.

All procedures with mice were approved by the Institutional Animal Care and Use Committee at the University of Oklahoma Health Sciences Center.

### Analysis of transcriptome

Transcriptome analysis was done using strand-specific RNA-Seq technology. For the RNA-Seq analysis, RNA samples were obtained from the animals fed DR for 1 month and then switched to AL for 2 months. Briefly, RNA was isolated from each tissue (epididymal white adipose tissue, liver, colon, hypothalamus, and hippocampus) using the RNeasy kit from Qiagen (Germantown MD, USA). RNA integrity was checked using the Bioanalyzer (Agilent), and only samples with RNA integrity numbers >8 used in either RNA-Seq or qPCR studies. Individual samples from each group were pooled (*n* = 5) for the RNA-Seq analysis and analyzed independently for qPCR. RNA was depleted of ribosomal RNA (Ribozero, Illumina) and used for generation of stranded RNA sequencing libraries, whereby the orientation and originating DNA strand of the RNA are maintained (Illumina Stranded RNA-Seq). Each library was uniquely indexed and then sized and quantified by capillary electrophoresis (TapeStation, Agilent). Libraries were sequenced in a paired-end 75 fashion on an Illumina HiSeq 2000 in rapid run mode. An average of 81.1 ± 9.5 million reads was generated for each library.

Raw fastq files were imported into Strand software (v9.5, Agilent) for trimming, alignment, and statistical analysis. Sequence with a Q score of <30 was discarded, 1 base from the 3′ and 5′, and adaptors sequences was removed. Reads were aligned to mm10 (November 26, 2014) in an orientation-specific fashion and duplicate reads were removed. Data was normalized for each tissue to the AL group for that tissue and quantitation performed with the DESeq algorithm (Anders and Huber 2010). A pairwise *Z* test was used to determine differential expression (*p* < 0.05) with Benjamini-Hochberg correction for the number of genes analyzed. For the *Z* test, reads were normalized by the total number of mapped reads. Assuming read counts to be distributed as Poisson, they were subjected to a square root transformation in an effort to make them nearly normally distributed with a constant variance and subjected to the *Z* test. Differentially expressed genes were further filtered to remove statistically significant changes with a fold change <|1.25|.

### Analysis of specific messenger RNA (mRNA) transcripts (RT-PCR)

The levels of specific mRNA transcripts of candidate genes (*Pomc*, *Crh*, *Hcrt*, *Foxg1*, *CSF2RB*, *Hsph1*, and *Ncoa7*) were measured in the individual samples (*n* = 5/group) with three technical replicates using real-time PCR. Briefly, RNA used for RNA-Seq was used for the RT-PCR analysis. The first-strand complementary DNA (cDNA) was synthesized from 1 μg RNA using random primers (Promega, Madison, WI, USA) and purified using the QIAquick PCR purification kit (Qiagen, Germantown, MD, USA). Expression of the candidate genes was quantified using real-time PCR with predesigned Taqman probes (Thermo Fisher, Waltham, MA, USA). Relative gene expression was quantified as comparative ct analysis using the 2^-ΔΔct^ analysis method with β-actin as endogenous control. One-way ANOVA design with Tukey’s multiple test correction was used to statistically analyze individual samples.

The levels of specific mRNA transcripts of three candidate genes identified from the RNA-Seq data (*Pomc*, *Hsph1*, and *Nts1*) were also measured on individual samples using real-time PCR on RNA samples obtained from animals fed DR for 4 months and then switched to AL for 5 months. RNA was co-isolated with DNA using the All-prep DNA/RNA co-isolation kit from Qiagen (Germantown MD, USA). The first-strand cDNA was synthesized from 1 μg RNA using random primers (Promega, Madison, WI, USA) and purified using the QIAquick PCR purification kit (Qiagen, Germantown, MD, USA). Expression of the genes was quantified using predesigned Taqman probes (Thermo Fisher, Waltham, MA, USA). Relative gene expression was quantified as comparative ct analysis using the 2^-ΔΔct^ analysis method with β-actin as endogenous control. One-way ANOVA design with Tukey’s multiple test correction was used to statistically analyze individual samples.

The assay number for each gene primer kit is as following: Mm00435874_m1 Pomc, Mm01293920_s1 Crh, Mm01964030_s1 Hcrt, Mm02059886_s1 Foxg1, Mm00655745_m1 Csf2rb, Mm00442864_m1 Hsph1, Mm00552797_m1 Ncoa7, Mm00481140_m1 Nts, and Mm02619580_g1 Actb.

### DNA methylation

The analysis of DNA methylation in the promoters of three genes used the second set of animals in which the mice were fed DR for 4 months and then switched to AL for 5 months. DNA/RNA was co-isolated using the All-prep DNA/RNA co-isolation kit from Qiagen (Germantown MD, USA). DNA methylation in the promoter regions of candidate genes (*Pomc*, *Hsph1*, and *Nts1*) was measured using the targeted BSAS technology, which provides highly accurate single-cytosine methylation quantitation in both CG and non-CG contexts. Briefly, genomic DNA was bisulfite converted and PCR amplified using specific primers designed using MethPrimer Express software corresponding to the genes to be analyzed. Primers were designed around sequences covering ~300–600 bp of the 5′ flanking region of each gene. The amplicons were purified and examined individually. Dual-indexed sequencing libraries were then made using the high-throughput tagmentation method [Nextera XT (Quail et al. 2012; Caruccio 2011)] and sequenced at great depth (>1,000×) to provide an accurate and reproducible quantitative analysis of cytosine methylation. Data analysis of the methylomes used workflow developed using Genomics Workbench software (Masser et al. 2013). One-way ANOVA design with Tukey’s multiple test correction was used to statistically analyze total 5mC sites. One-way ANOVA design with Benjamini-Hochberg correction was used to statistically analyze individual 5mC sites.

## Results

### Effect of short-term DR on gene expression

**Fig. 1:**
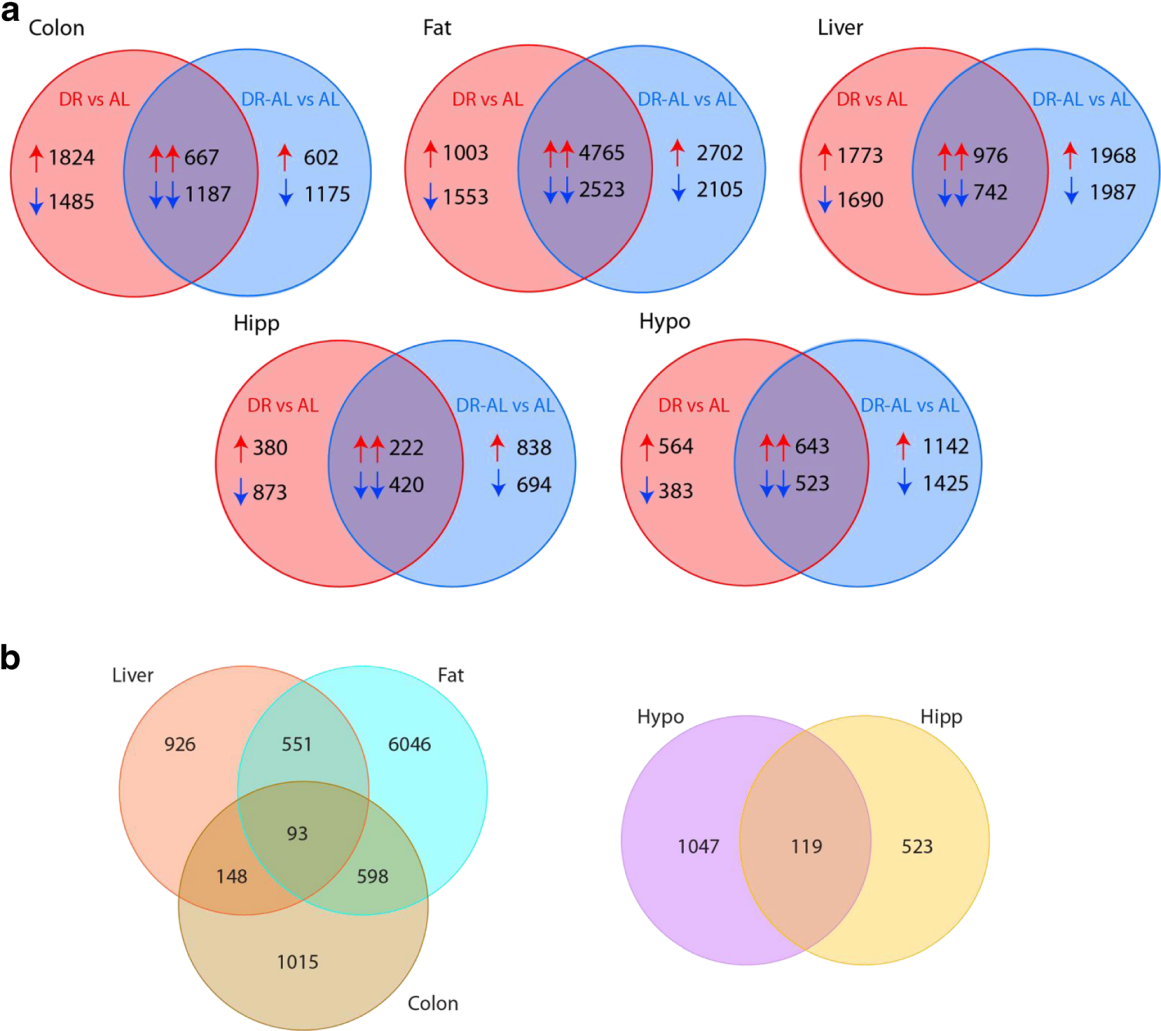
Transcriptome analysis of mice fed DR for 1 month and then switched to AL for 2 months. **a** Total number of genes are shown that were altered with 1 month DR (DR) and persisted after switching DR to AL (DR-AL) both normalized to AL. Data were obtained from pooled samples (*n* = 5/group) using RNA-Seq. The *single arrows* indicate the number of genes upregulated or downregulated in either DR or DR-AL, while the *double arrows* in the region of the *circles* that overlap indicate number of genes that remained upregulated or downregulated when switched to AL (DR-AL). **b** Common genes identified between tissues that showed persistent expression when switched from DR to AL. The genes found in the overlap (from **a)** for each tissue were further analyzed to identify common genes between tissues. Neurological (hippocampus and hypothalamus) and non-neurological (liver, fat, colon) tissues are shown separately. As described in the methods, we statistically compared the data from the pooled samples by normalizing the total mapped reads generated in each condition and transforming them to a normal distribution with constant variance and then analyzing the data with a *Z* test to do pairwise comparisons as hypothesis discovery (fold change >1.25)

Since the first study by our group in 1987 showing that DR altered the transcription of specific genes (Richardson et al. 1987), there have been a large number of additional studies showing that DR alters the transcription of genes in a variety of tissues (Lee et al. 1999). However, all of the previous studies have measured the effect of DR on the levels of mRNA transcripts after long-term DR, e.g., when mice or rats were relatively old and had been maintained on DR for 1 to 2 years. In other words, there is essentially no information on whether short-term DR alters gene expression. We hypothesized that if DR had a memory effect, DR would alter gene expression within a few months, and the alterations in at least some of the genes would persist when DR was discontinued. To test our hypothesis, we fed male C57BL/6 mice a DR diet (60% AL) for 1 month and then fed them AL for 2 months. We used RNA-Seq analysis as an unbiased approach to compare gene expression in five different tissues (epididymal white adipose tissue, liver, colon, hypothalamus, and hippocampus) from pooled samples of mice fed AL, DR, and DR switched to AL (DR-AL). We chose these tissues because they are some of the most sensitive tissues with respect to changes in the diet. For example, the hypothalamus is the primary organ responsible for sensing changes in food consumption, integrating/coordinating the various signals in response to altered nutrient intake, and relaying this information to tissues throughout the body. It has also been argued that the hypothalamus plays an important role in DR’s antiaging action, e.g., Minor et al. (2011) showed that lesions in the ARC of the hypothalamus blocks the tumor suppression effect of DR and the reduction of fasting blood glucose levels, which are observed in DR mice. DR has been shown to be neuroprotective in the hippocampus by upregulating genes such as BDNF, klotho, and transthyretin (Kishi and Sunagawa 2012; Schafer et al. 2015). DR has also been shown to have a beneficial impact on the white adipose tissue and liver, which are at the crux of energy metabolism, e.g., DR has been shown to improve insulin sensitivity and glucose metabolism via its effect on adipose tissue and liver (Yu et al. 2014). DR also has a significant impact on colon, protecting the tissue from the development of colon cancer (Kumar et al. 1990; Olivo-Marston et al. 2014) and preserving and enhancing the self-renewal capacity of intestinal stem cells by increasing their function and proliferation (Yilmaz et al. 2012). As shown in Fig. 1a, we found a substantial number of mRNA transcripts that were either upregulated or downregulated in expression across the five tissues after only 1 month of DR compared to mice fed AL. More importantly, we observed a large number of these genes in the five tissues continued to remain changed after discontinuing DR and feeding the mice AL for 2 months. For example, ~15–56% of the mRNA transcripts remained upregulated, and ~17–41% of the mRNA transcripts remained downregulated in total in all tissues when DR was discontinued and the mice were fed AL. For some tissues, such as fat, the number of persistent genes was very high (~50%), whereas the liver, colon, hippocampus, and hypothalamus showed ~19–27% changes in gene expression which persisted when the mice were fed AL. Further, when we analyzed the genes found to be persistent even after switching to AL, we found common genes across the tissues (Fig. 1b). As shown in Fig. 1b, we found 93 of the persistent genes to be common among the liver, fat, and colon and 119 of the persistent genes to be common between hypothalamus and hippocampus. Taken together these data, which are limited because they are from pooled samples, support our hypothesis that DR has a memory effect and could potentially alter gene expression through DNA methylation.

**Fig. 2:**
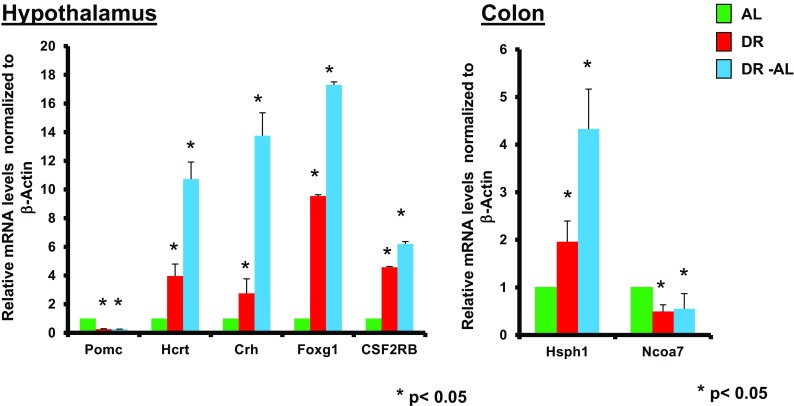
Expression levels of specific mRNA transcripts in tissues from mice fed DR for 1 month (DR) and then switched to AL for 2 months (DR-AL). The expression of seven of the genes (*Pomc*, *Crh*, *Hcrt*, *Foxg1*, *CSF2RB*, *Hsph1*, and *Ncoa7*) in hypothalamus and colon that showed significant alterations through the RNA-Seq measurements were confirmed using real-time PCR. The levels of the mRNA transcripts are the mean ± SEM of data from five mice per group and were statistically analyzed by one-way ANOVA with Tukey’s multiple test correction. The *asterisk* indicates that the values for the DR and DR-AL mice are significantly different (*P* < 0.05) from the AL mice

To confirm the changes in expression in individual samples (*n* = 5), we measured the expression of a subset of seven genes that showed significant differences in their expression (from RNA-Seq data) using real-time PCR (Fig. 2). It should be noted that there are other genes that fit these parameters; however, these genes were selected because they showed robust changes with short-term DR and might play a role in the mechanism of DR. In the hypothalamus, we found that the expression of *Pomc* (proopiomelanocortin) was reduced over 65%, while the expression of *Crh* (corticotrophin releasing hormone), *Hcrt* (hypocretin), *Foxg1* (forkhead box G1), and *Csf2rb* (colony stimulating factor 2 receptor beta) were increased twofold to eightfold after 1 month of DR. In the colon, we observed a twofold increase in the expression of *Hsph1* (heat shock protein family H member 1) and a ~50% decrease in the expression of *Ncoa7* (nuclear coactivator 7). More importantly, we found that the changes in expression (either up or down) induced by 1 month of DR were maintained after feeding the mice AL for 2 months. In fact, several of the genes showed an even greater change in expression when switched to AL. As seen in Fig. 2, all genes shown to increase with DR in the hypothalamus (*Hcrt*, *Crh*, *Foxg1*, *Csf2rb*) and colon *(Hsph1*) demonstrated an even greater increase in the level of expression when switched to AL, exhibiting a further twofold to sevenfold increase in expression.

### Effect of DR on DNA methylation of specific genes

Because the data in Figs. 1 and 2 show that short-term DR can alter the expression of specific genes by a mechanism that persisted after the mice were returned to AL feeding, we measured the expression and DNA methylation in the promoter region of three specific genes in either the hypothalamus (*Pomc*) or colon (*Hsph1* and *Nts1*) in a second group of animals: mice fed either AL, DR for 4 months, or DR for 4 months and then AL for 5 months (DR-AL). We measured DNA methylation using the BSAS assay, which gives high quantitative accuracy and reduced technical variance because of the ultra-deep level of sequencing the assay provides (Masser et al. 2013). Using BSAS assay, we measured the DNA methylation status of cytosines, in both CG and CH (where H is either A, T, or C) contexts, found in the cis-acting elements of the promoter regions of candidate genes whose expression was found to be significantly altered by DR and remained altered when DR was discontinued; two genes in the colon (*Hsph1* and *Nts1*) and one gene in the hypothalamus (*Pomc*).

**Fig. 3:**
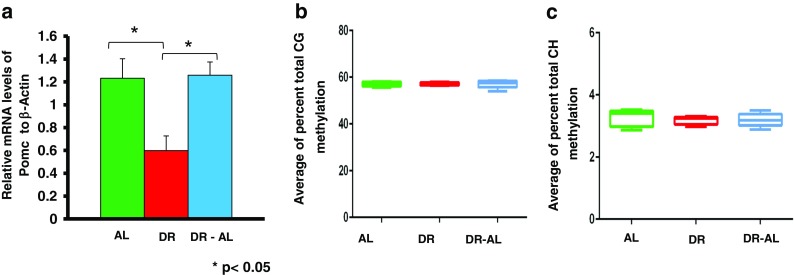
Effect of DR on DNA methylation and expression of *Pomc* gene in hypothalamus from mice fed either AL, DR for 4 months (DR), or switched from DR to AL for 5 months (DR-AL). **a** Expression of *Pomc* mRNA measured using real-time PCR. **b** The percent of total CG methylation in the 560 bp promoter of the *Pomc* gene as measured by BSAS are shown as box plots. **c** The percent of total CH methylation in the 560 bp promoter of the *Pomc* gene as measured by BSAS are shown as box plots. The mRNA expression data are the mean ± SEM of data from five mice per group and were statistically analyzed by one-way ANOVA with Tukey’s multiple test correction. The DNA methylation data are the mean ± SEM of data from five mice per group and were statistically analyzed by one-way ANOVA with Tukey’s multiple test correction for the total 5mC sites. The *asterisk* indicates that the values for the DR and DR-AL mice are significantly different (*P* < 0.05) from the AL mice

**Fig. 4:**
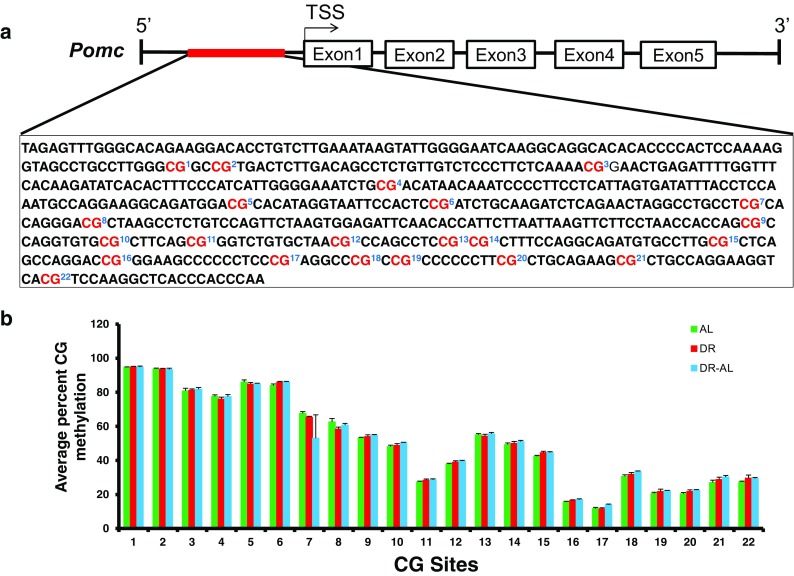
Effect of DR on the methylation of specific cytosines in the promoter of the *Pomc* gene in the hypothalamus from mice fed either AL, DR for 4 months (DR), or switched from DR to AL for 5 months (DR-AL). **a** The location of the 22 CG sites in the 560 bp of 5′ flanking region of the *Pomc* gene. **b** The level of cytosine methylation at the 22 CG sites. The methylation data are the mean ± SEM of data from five mice per group and were statistically analyzed by one-way ANOVA with Benjamini-Hochberg correction for the individual sites

We first measured the expression and methylation status of the *Pomc* promoter in the hypothalamus. Pomc is expressed by the Pomc neurons of the arcuate nucleus of the hypothalamus (Woods et al. 2008). Its expression has been shown to be regulated by DNA methylation, e.g., overfeeding and high fat feeding have been shown to result in hypermethylation and reduced expression of the *Pomc* gene in the hypothalamus (Plagemann et al. 2009; Marco et al. 2013), and obese men who regain weight after stopping dieting show hypermethylation of the promoter of the *Pomc* gene (Crujeiras et al. 2013). As shown in Fig. 3a, *Pomc* mRNA levels were significantly reduced by 4 months of DR, which is similar to what we observed after 1 month of DR (Fig. 2). However, in contrast to what we observed after 2 months of feeding AL (Fig. 2 **)**, *Pomc* mRNA levels rebounded to levels similar to that observed in mice fed AL when the DR mice were fed AL for 5 months. We analyzed the methylation status of the CG and CH sites found in the 5′-flanking region (560 bp region) of the *Pomc* promoter (Fig. 3b, c). Although we observed a high level of total CG and CH methylation, we did not observe any significant difference in total DNA methylation in this region of the *Pomc* promoter between the DR and DR-AL groups compared to their AL counterparts. The 560 bp region of the *Pomc* promoter studied has 22 individual CG sites and 136 individual CH sites, and Fig. 4 shows the level of methylation at each of the 22 CG sites. Again, we observed no significant differences in the level of methylation at any of the 22 CG sites (Fig. 4) and 136 CH sites (data not shown) between the three groups studied (AL, DR, and DR-AL). Thus, the changes in *Pomc* expression induced by DR do not arise from changes in the methylation status in the 560 bp region of the *Pomc* promoter.

**Fig. 5:**
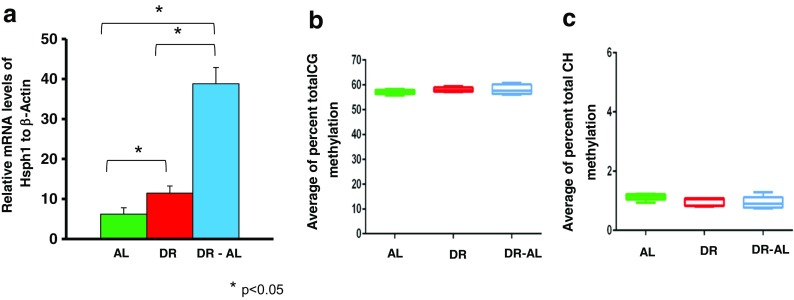
Effect of DR on DNA methylation and expression of *Hsph1* gene in the colon from mice fed either AL, DR for 4 months (DR), or switched from DR to AL for 5 months (DR-AL). **a** Expression of *Hsph1* mRNA measured using real-time PCR. **b** The percent of total CG methylation in the 342 bp promoter of the *Hsph1* gene as measured by BSAS are shown as box plots. **c** The percent of total CH methylation in the 342 bp of the promoter of the *Hsph1* gene as measured by BSAS are shown as box plots. The mRNA expression data are the mean ± SEM of data from five mice per group and were statistically analyzed by one-way ANOVA with Tukey’s multiple test correction The DNA methylation data are the mean ± SEM of data from five mice per group and were statistically analyzed by one-way ANOVA with Tukey’s multiple test correction for the total 5mC sites. The *asterisk* indicates that the values for the DR and DR-AL mice are significantly different (*P* < 0.05) from the AL mice

**Fig. 6:**
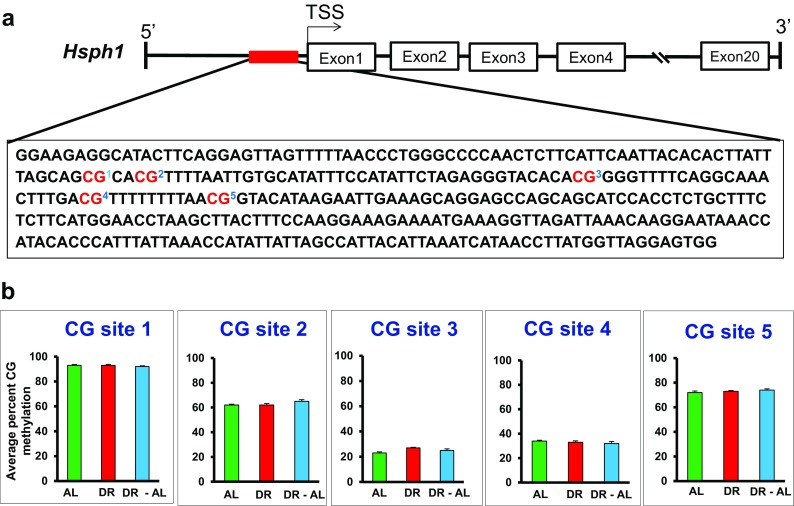
Effect of DR on the methylation of specific cytosines in the promoter of the *Hsph1* gene in the colon from mice fed either AL, DR for 4 months (DR), or switched from DR to AL for 5 months (DR-AL). **a** The location of the 5 CG sites in 342 bp of 5′-flanking region of the *Hsph1* gene. **b** The level of cytosine methylation at the 5 CG sites. The methylation data are the mean ± SEM of data from five mice per group and were statistically analyzed by one-way ANOVA with Benjamini-Hochberg correction for the individual sites

We next studied the *Hsph1* gene in the colon because *Hsph1* expression was significantly increased with DR, and the increase was even more pronounced when the diet was switched from DR to AL (Fig. 5a). We analyzed the methylation status of the CG/CH sites found in the ~342 bp region of the promoter (Fig. 5b, c). We found a high level of CG and CH methylation in the promoter region, but there was no significant difference in total DNA methylation between the groups. We also measured the methylation of the 5 specific CG sites and 63 CH sites in the ~342 bp region of the *Hsph1* promoter (Fig. 6). Neither CG sites nor CH sites (data not shown) showed any significant difference in methylation between the three groups of mice. Therefore, the methylation of the CG/CH sites in this region of the *Hsph1* promoter is not involved in the increased expression of the *Hsph1* gene induced by DR.

**Fig. 7:**
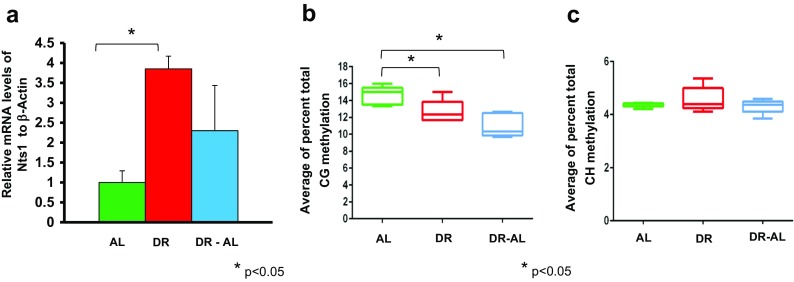
Effect of DR on DNA methylation and expression of *Nts1* gene in the colon from mice fed either AL, DR for 4 months (DR), or switched from DR to AL for 5 months (DR-AL). **a** Expression of *Nts1* mRNA measured using real-time PCR. **b** The percent of total CG methylation in the 397 bp promoter of the *Nts1* gene as measured by BSAS are shown as box plots. **c** The percent of total CH methylation in the 397 bp promoter of *Nts1* gene as measured by BSAS are shown as box plots. The mRNA expression data are the mean ± SEM of data from five mice per group and were statistically analyzed by one-way ANOVA with Tukey’s multiple test correction. The DNA methylation data are the mean ± SEM of data from five mice per group and were statistically analyzed by one-way ANOVA with Tukey’s multiple test correction for the total 5mC sites. The *asterisk* indicates that the values for the DR and DR-AL mice are significantly different (*P* < 0.05) from the AL mice

**Fig. 8:**
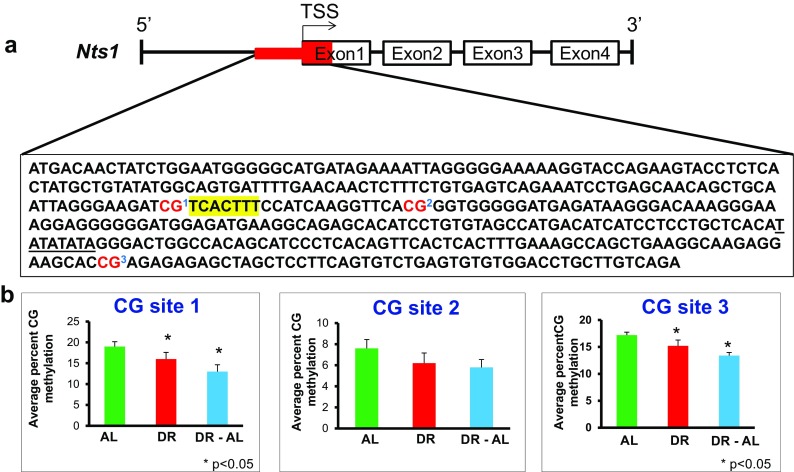
Effect of DR on the methylation of specific cytosines in the promoter of the *Nts1* gene in the colon from mice fed either AL, DR for 4 months (DR), or switched from DR to AL for 5 months (DR-AL). **a** The location of the 3 CG sites in 397 bp of 5′-flanking region overlapping TSS of the *Nts1* gene. The distal AP-1 consensus site is *highlighted* and the TATA box is *underlined*. **b** The level of cytosine methylation at the 3 CG sites. The methylation data are the mean ± SEM of five mice per group and were statistically analyzed by one-way ANOVA with Benjamini-Hochberg correction for the individual sites. The *asterisk* indicates that the values for the DR and DR-AL mice are significantly different (*P* < 0.05) from the AL mice

We next looked at the expression and methylation status of the *Nts1* gene in the colon. Nts1 expression has been shown to be differentially regulated in human colon cancer cell lines by changes in DNA methylation (Dong et al. 2000). As shown in Fig. 7a, DR increased the expression of *Nts1* after 4 months of DR, and *Nts1* expression continued to show an increasing trend even when DR was discontinued for 5 months. Interestingly, we found significant differences in the total CG methylation in the 5′-flanking region of the *Nts1* gene, i.e., the 397 bp region of the promoter overlapping the TSS and 5′-end of exon1, which contains the distal AP-1 consensus site (Fig. 7b). The overall DNA methylation in the *Nts1* promoter region was significantly reduced (~14% percent decrease) by DR. The hypomethylation persisted even when DR was discontinued and was correlated with *Nts1* mRNA expression. The total CH methylation remained unaltered after DR (Fig. 7c). The data in Fig. 8 shows the methylation of the 3 CG sites found in the 397 bp region of the *Nts1* promoter. All three sites showed hypomethylation with DR, and this hypomethylation persisted even when DR was discontinued. The percent decrease in DNA methylation ranged from 12 to 16% with DR and 22–32% when switched to AL. This decrease in methylation was significant for CG sites 1 and 3, the two sites with the highest level of methylation in the AL mice. No significant difference was observed in methylation in any of the 75 CH sites in the 5′-flanking region of the *Nts1* gene (data not shown).

**Fig. 9:**
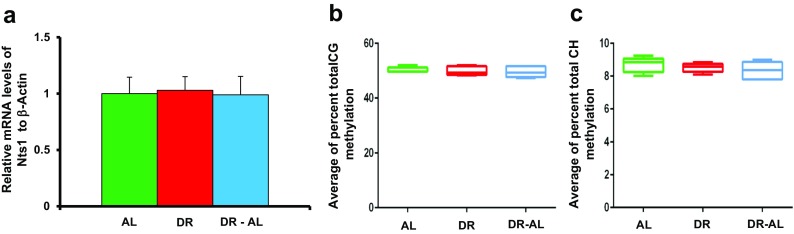
Effect of DR on DNA methylation and expression of *Nts1* gene in the hypothalamus from mice fed either AL, DR for 4 months (DR), or switched from DR to AL for 5 months (DR-AL). **a** Expression of *Nts1* mRNA measured using real-time PCR. **b** The percent of total CG methylation in the 397 bp promoter of *Nts1* gene as measured by BSAS are shown as box plots. **c** The percent of total CH methylation in the 397 bp promoter of *Nts1* gene as measured by BSAS are shown as box plots. The mRNA expression data are the mean ± SEM of data from five mice per group and were statistically analyzed by one-way ANOVA with Tukey’s multiple test correction. The DNA methylation data are the mean ± SEM of data from five mice per group and were statistically analyzed by one-way ANOVA with Tukey’s multiple test correction for the total 5mC sites

**Fig. 10:**
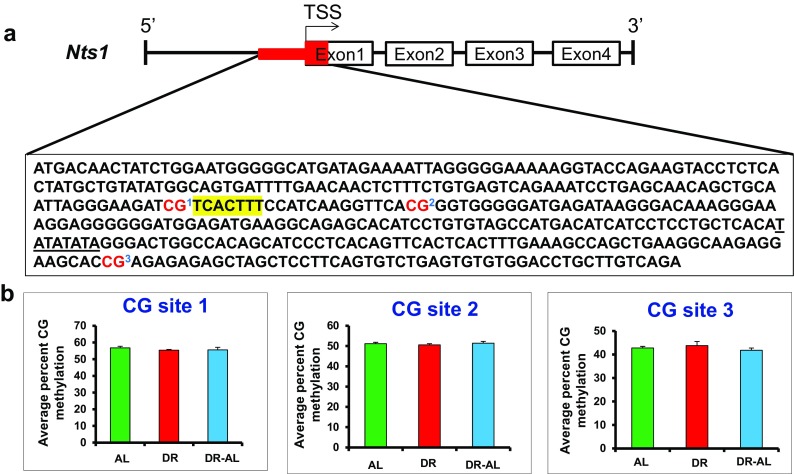
Effect of DR on the methylation of specific cytosines in the promoter of the *Nts1* gene in the hypothalamus from mice fed either AL, DR for 4 months (DR), or switched from DR to AL for 5 months (DR-AL). **a** The location of the 3 CG sites in 397 bp of 5′ flanking region overlapping TSS of the *Nts1* gene. The distal AP-1 consensus site is *highlighted* and TATA box is *underlined*. **b** The level of cytosine methylation at the 3 CG sites. The methylation data are the mean ± SEM of data from five mice per group and were statistically analyzed by one-way ANOVA with Benjamini-Hochberg correction for the individual sites

Because we observed that DR induced changes in the methylation status of the *Nts1* promoter in the colon correlated with the increased expression of *Nts1* mRNA, we measured the expression and DNA methylation of the *Nts1* gene in the hypothalamus. *Nts1* is a neuropeptide first identified in the hypothalamus (Carraway and Leeman 1973). *Nts1* did not show any significant difference in its expression in the hypothalamus of AL, DR, and DR-AL mice (Fig. 9a). Although the *Nts1* gene promoter has a high level of methylation in the hypothalamus when compared to colon, neither the total CG/CH methylation nor the methylation of individual CG sites showed a significant difference between the three groups studied (Figs. 9 and 10).

## Discussion

It is becoming increasingly apparent that environmental/nutritional factors can induce epigenetic changes (e.g., DNA methylation and histone modification), which can potentially result in long-term changes in gene expression and therefore the function of cells and tissues in an organism. Recent studies have generated a great deal of interest in the possible role that DNA methylation might play in human aging, e.g., several groups showed that DNA methylation at specific CG sites was a quantitative chronological biomarker of human aging (Hannum et al. 2013; Horvath 2013; Weidner et al. 2014), and Melov’s group showed that changes in DNA methylation occurred at a number of genomic loci with age in human skeletal muscle that appeared to be associated with changes in gene expression (Zykovich et al. 2014). Because DR is the most studied manipulation to increase longevity and retard aging in animals ranging from invertebrates to mammals, it is not surprising that epigenetics has been proposed to play a role in the antiaging mechanism of DR (Li et al., 2011). The observations that short-term DR can increase the lifespan of mice (Yu et al. 1985; Sun et al. 2009) as well as biological functions such as insulin sensitivity (Cameron et al. 2012; Selman and Hempenstall 2012) demonstrate that DR has a “memory” effect that can persist even after DR is discontinued, a hallmark characteristic of an epigenetic mechanism.

Currently, there are only limited data on the effect of DR on DNA methylation. In an early study, Hass et al. (1993) used restriction enzymes to compare the methylation in the *Ras* gene of pancreatic acinar cells from old AL and DR rats. They stated (no data were provided) that the DNA of the *Ras* gene was hypomethylated in the cells from rats fed AL compared to DR rats and was correlated with the increased expression of the *Ras* gene in the DR rats. Chouliaras et al. (2011) reported that DR attenuated the age-related changes in a DNA methyltransferase (Dnmt3a) in the hippocampus of mice, though we have recently demonstrated a lack of Dnmt3a regulation with aging in the hippocampus (Hadad et al. 2016). Two recent studies suggest that DR can alter DNA methylation. Li et al. (2010) reported that glucose restriction in WI-38 cells induced lifespan extension and decreased expression of p16, which were correlated to an increase in DNA methylation and decreased active chromatin markers (e.g., acetyl-H3, acetyl-H4, and dimethyl-H3) in the *p16* promoter region which was identified as putative E2F-1 binding site an active transcription factor of p16. Kim et al. (2016) showed that 4 weeks of DR in old rats resulted in >100,000 differentially methylated regions in DNA from the kidney using the MeDIP-Seq assay, which provides a relative, not absolute quantitation of 5mC levels. While this study showed that methylation in promoter and intron regions decreased in old rats and were increased in old DR rats, these changes in DNA methylation induced by DR altered were not correlated with the expression of any specific genes, i.e., the functional consequences of these changes in DNA methylation are not known.

We rationalized that if epigenetics plays a role in DR’s mechanism of action, short-term DR should alter the transcriptome in a variety of tissues and the changes in the expression of at least some genes would persist when DR is discontinued and animals are fed AL. Using RNA-Seq, we showed that within 1 month of DR, there were changes in the expression of a large number of genes in all five tissues studied, and more importantly, we found that ~19–50% of these changes persisted in total when DR was discontinued and the animals were fed AL for 2 months. This is the first study to show that major changes in the transcriptome occurred within a month of DR, and that many of the changes in gene expression induced by DR were maintained when mice are returned to AL feeding.

We next determined whether short-term DR (4 months) altered DNA methylation in specific promoter regions that would persist after switching mice to AL for 5 months and whether the changes in methylation would correlate to changes in gene expression. We measured the methylation in the 5′ flanking regions (400–560 bp of the promoters) adjacent to the transcription start site and the 5′-end of exon1 of three genes using the BSAS assay, which allowed us to absolutely quantify methylation with base-specific resolution. We first focused on the *Pomc* gene in the hypothalamus because the hypothalamus is the primary organ responsible for sensing changes in food consumption, integrating/coordinating the various signals in response to altered nutrient intake, and relaying this information to tissues in the whole body (Woods et al. 2008; Dacks et al. 2013) and because the expression of Pomc in response to nutritional manipulations has been shown to be regulated by DNA methylation (Plagemann et al. 2009; Marco et al. 2013). We did not observe any significant change with DR in any of the 22 CG sites or in the 136 CH sites in the 560 bp promoter region of the *Pomc* gene. This region of the *Pomc* promoter contained the CG sites that had been previously shown to be altered by nutritional manipulations (Plagemann et al. 2009; Crujeiras et al. 2013; Marco et al. 2013). Therefore, it is possible that DR does not affect DNA methylation in the hypothalamus as do other nutritional manipulations. It is also possible that DNA methylation in the *Pomc* promoter was altered by DR only in the cells in the arcuate nucleus, the region of the hypothalamus that expresses Pomc, because these cells would make up a small minority of the cells in the whole hypothalamus, and therefore, changes in methylation might be masked by the majority of other cells that do not express Pomc.

We also measured DNA methylation of two genes in the colon (intestinal mucosa) from mice fed DR: *Hsph1* and *Nts1*. Again, we found no effect of DR on methylation of any of the 5 CG sites or the 63 CH sites in the *Hsph1* promoter, even though DR induced Hsph1 expression ~twofold. In contrast, we observed a decline in DNA methylation at the 3 CG sites in the *Nts1* promoter, encompassing the distal AP-1 consensus site, which was inversely correlated with increased Nts1 mRNA levels. A significant (~12–16%) percent decrease in DNA methylation was observed in two of the three sites, and the percent decreased methylation was more pronounced (~22–32%) after the DR mice were fed AL for 5 months. In contrast, DR had no effect on either Nts1 expression or the methylation of the *Nts1* promoter in the hypothalamus. Thus, we observed a strong correlation between the changes in DNA methylation in the *Nts1* promoter and the expression of Nts1 suggesting that the changes in Nts1 expression induced by DR in the colon occurs at least partially through alterations in DNA methylation. It is important to note that Dong et al. (2000) showed hypomethylation of the *Nts1* promoter at the same 3 CG sites induced Nts1 expression in KM12C cells, whereas hypermethylation of these 3 CG sites silenced the *Nts1* gene in the KM20 cell line. These data demonstrate, as expected, DR has a tissue and gene specific epigenetic effect.

The mucosa cells we isolated from the colon are composed mostly of epithelial cells, which are continuously renewed every 4 to 5 days. Therefore, it is likely that the changes in DNA methylation induced by DR occur in the stem cells that reside in the crypt base of the intestinal glands and give rise to the epithelial cells. In line with this concept is the report by Sheaffer et al. (2014) showing that DNA methylation is required for the control of intestinal stem cell differentiation and that loss of DNA methylation, which was achieved by conditional ablation of *Dnmt1* in the intestine, leads to intestinal crypt expansion by delaying differentiation and increasing the proliferative capacity of stem cells. Interestingly, Yilmaz et al. (2012) showed that short-term (~6 months) DR (60% AL) enhanced the proliferative and self-renewal capacity of the intestinal stem cells in mice but reduced the number of differentiated cells. Although Yilmaz et al. (2012) did not explore the potential mechanism(s) behind this shift in intestinal stem cells, changes in DNA methylation induced by DR in the *Nts1* gene could be important because Nts1 is a gut tridecapeptide that acts as a potent cellular mitogen (Wang et al. 2006). Thus, the increased expression of Nts1, which we have observed, could play a role in the ability of DR to enhance the proliferation and reduce differentiation of intestinal cells observed by Yilmaz et al. (2012). These changes in proliferation and differentiation would be predicted to preserve and increase the intestinal stem cell pool in the DR mice.

In summary, our data are consistent with the view that DNA methylation plays a role in the memory effect observed in DR. The expression of Nts1 induced by short-term DR in the colon was strongly correlated with methylation at specific CG sites within the promoter of *Nts1* gene, e.g., methylation was decreased by DR and remained reduced when the mice were fed AL for 5 months, and DR had no effect on either Nts1 expression or DNA methylation in the promoter of the *Nts1* gene in the hypothalamus. Thus, our data are consistent with the possibility that short-term DR can induce changes in DNA methylation at specific CG sites in the promoter of a gene that persists after DR is discontinued and are correlated with changes in the expression of that gene. In addition, our data suggest that the changes in Nts1 expression might be functionally important by playing a role in preservation of the intestinal stem cell pool. Our data also show that the effect of DR on DNA methylation is both gene and tissue specific. In other words, the changes in DNA methylation induced by DR most likely do not involve changes in the total levels/activities of proteins involved in DNA methylation, e.g., DNA methyltransferases and demethylases such as TET methylcytosine dioxygenases. Thus, our data demonstrate the need for investigators to quantify methylation with base specificity/single-base resolution throughout the genome of various tissues when evaluating the role of DNA methylation in the biological mechanism of DR.
